# A novel α-fetoprotein-derived helper T-lymphocyte epitope with strong immunogenicity in patients with hepatocellular carcinoma

**DOI:** 10.1038/s41598-020-60843-4

**Published:** 2020-03-04

**Authors:** Toshikatsu Tamai, Eishiro Mizukoshi, Masashi Kumagai, Takeshi Terashima, Noriho Iida, Masaaki Kitahara, Tetsuro Shimakami, Kazuya Kitamura, Kuniaki Arai, Taro Yamashita, Yoshio Sakai, Tatsuya Yamashita, Masao Honda, Kazumi Fushimi, Shuichi Kaneko

**Affiliations:** Department of Gastroenterology, Graduate School of Medicine, Kanazawa University, Kanazawa Ishikawa, Japan

**Keywords:** Cancer, Immunology

## Abstract

α-Fetoprotein (AFP) is considered a good target for immunotherapy strategies against hepatocellular carcinoma (HCC); however, no immunodominant AFP-derived MHC class II-restricted helper T-lymphocyte (HTL) epitope has been reported. Therefore, we identified novel AFP-derived HTL epitopes possessing high immunogenicity. HTL epitopes were predicted using the online service, and peptides were subsequently synthesized. Four newly synthesized peptides showed positive reactivity in >20% patients on ELISPOT using peripheral blood mononuclear cells (PBMCs). Among these, the highest rate was shown by AFP_1_ (MKWVESIFLIFLLNFTESRT), which also showed the highest positive rate in cell proliferation assays. Binding assays demonstrated that AFP_1_ had strong binding properties toward MHC molecules. Further, blocking assays performed using an anti-HLA-DR antibody showed that immune response decreased, confirming the binding of AFP_1_ to HLA-DR molecules. Furthermore, the survival rates of patients with stages II–IV HCC indicated that T cell response against AFP_1_ led to significantly greater survival that of patients without T cell response. When evaluating immune response against AFP_1_ before and after HCC treatment, an increase in the frequency of peptide-specific T cells was observed after treatment in patients with HLA-DRB1*1502, *0405, and *0901 alleles. In conclusion, the identified epitopes may be useful for immunotherapy strategies against HCC.

## Introduction

Among the different types of cancer, liver cancer is the second and sixth most common cause of death in males and females, respectively, worldwide^1^. Hepatocellular carcinoma (HCC) accounts for 70%–90% of primary liver cancers^1^. Chronic liver injury and liver cirrhosis are the major factors considered responsible for the development of liver cancer^2^. Current treatments for early-stage HCC include resection, transplantation, and locoregional therapy. However, the high rate of post-treatment relapse remains a challenge. Moreover, limited options are available for the treatment of advanced stage HCC^3,4^, with the exception of systemic therapies such as sorafenib, regorafenib, and lenvatinib^5–7^. The drug cabozantinib has been shown to prolong the survival period in HCC^8^; however, in general, these drugs prolong survival by only a few months.

In recent years, treatments using immune checkpoint inhibitors (ICIs) and chimeric antigen receptor-modified T cells have been clinically used in cancer immunotherapy and have shown positive therapeutic efficacies. Indeed, recently developed cancer immunotherapy strategies, which substantially differ from current cancer treatments, represent an innovative approach because they involve the host immune system as the target. Thus, novel cancer treatment regimens are being developed as a paradigm shift. Furthermore, the importance of recognizing the antigens expressed by cancer cells as targets and the removal of those cancer cells by T cells has been highlighted by the success of these immunotherapy treatments. Accordingly, research to identify highly immunogenic T cell epitopes that recognize such antigens, including neoantigens and T cell receptors, is underway.

Clinical trials are ongoing for several ICIs (anti-PD-1, PD-L1, and CTLA-4 antibodies) for HCC treatment; however, reports have indicated that the overall response rate is approximately 20%, with the efficacies of the treatment strategies using ICI alone considered insufficient. Therefore, the development of combination therapies, such as combinations of one ICI with another ICI, molecular target drug, or other immune therapy, has garnered increasing attention.

AFP is a cancer-related antigen frequently expressed in HCC and used as a marker for HCC diagnosis and therapeutic efficacy. As such, the expression of AFP in healthy individuals is believed to be rare; therefore, the antigen is considered a promising target for cancer immunotherapy^9^. Among the available immunotherapies for liver cancer using AFP as a target, therapies using dendritic cells (DCs) stimulated with AFP-derived epitopes and vaccine therapies by class I-restricted peptides have been developed. However, these therapies have not shown sufficient efficacy in clinical settings^10,11^.

We previously reported on AFP-derived major histocompatibility complex (MHC) class I-restricted epitopes^12^. In this previous study, we treated HCC patients with an AFP peptide vaccine against the peptide and successfully obtained specific TCRs from the patients who positively responded^13^. These results indicated the efficacy of immunotherapy strategies using epitopes with strong immunogenicity for HCC. On the other hand, cases with limited efficacy indicated the necessity of an improved strategy to improve overall treatment efficacy. In recent years, it has been demonstrated that the function of helper T lymphocytes (HTLs) is important for increasing the therapeutic efficacy of immunotherapy strategies, in addition to that of cytotoxic T lymphocytes (CTLs). HTLs are believed to contribute to cancer therapy by increasing the reactivity of CTLs^14,15^. In fact, clinical trials using wild-type 1 peptide-pulsed DCs have indicated that activation of both CTLs and HTLs results in a better clinical response compared with the CTL vaccine alone^16^.

To date, several studies have reported on AFP-derived MHC class II-restricted epitopes. However, the identification of immunodominant epitopes with strong immunogenicity has not been reported^17–19^. Therefore, in this study, we identified and characterized novel AFP-derived MHC class II-restricted epitopes.

## Materials and Methods

### Patient population

Samples were collected from 47 patients (39 males and 8 females) aged 40 to 81 years (median: 68 years). Of these, 36 patients had HCC and 11 patients had a history of liver cancer. These patients regularly visited the Department of Gastroenterology at Kanazawa University Hospital (Kanazawa, Japan) between 2007 and 2013. Blood samples were collected from 36 cancer-bearing patients before treatment. In addition, blood samples were collected after treatment (3 days to 42 days) in 5 patients to compare responses before and after treatment. Eleven non-cancerous patients had a period of 0.6–64 months after the previous treatment at the time of blood collection. After receiving consent, samples were collected from 14 healthy adult individuals (Table 1). The diagnosis of HCC was based on typical patterns, such as hyperattenuation areas in the early phase and hypoattenuation areas in the late phase, by dynamic CT or MRI. We performed CT scan (from chest to pelvis), upper gastrointestinal endoscopy, and colonoscopy to confirm that there were no other malignancies. We further confirmed that the healthy individuals had no history of malignant tumors and were negative for the hepatitis B virus surface antigen (HBsAg) and HCV antibody. All methods were carried out in accordance with relevant guidelines and regulations. According to the Helsinki Declaration, the study protocol was explained to the subjects and informed consent was obtained from all who wished to participate. This study was approved by the regional ethics committee (Medical Ethics Committee of Kanazawa University, Protocol #829).

**Table 1:** Characteristics of the patients studied.

Clinical diagnosis	Patient No.	Sex, M/F	Age, years	ALT, IU/L	Lymph cell, /dL	AFP, ng/mL	Child-Pugh, A/B/C	Tumor size, large/small	Tumor multiplicity, multiple/solitary	Sstage, I/II/III/IV
Tumor-bearing patients	36	31/5	68 (40–81)	37.5 (10–159)	1005 (380–2100)	27 (6–40550)	31/3/1^a^	11/25	24/12	8/16/5/7
Previous treatment subjects	11	8/3	66 (54–80)	42 (10–89)	1140 (450–2520)	11 (4–139)	9/2/0	4/7	2/9	6/4/1/0
Normal Donors	14	12/2	32 (28–38)	14 (11–23)	ND	ND	ND	ND	ND	ND

Data are shown as the median (range); Large, >2 cm; small, ≤2 cm; ND, not determined.

^a^One patient on anticoagulant medication was excluded.

### Laboratory and virological testing

Blood samples were tested for the HBsAg and HCV antibody using commercial assay kits (Fuji Rebio, Tokyo, Japan). For human leukocyte antigen (HLA) typing, peripheral blood samples collected from the patients and healthy individuals were outsourced for analysis (BMI Inc., Tokyo, Japan) and confirmed using PCR. Serum AFP levels were measured by EIA (AxSYM AFP, Abbott Japan, Tokyo, Japan).

### Synthetic peptides

Amino acid sequences of AFP (GenBank accession number AAB58754)-derived MHC class II-binding HTL epitopes were predicted using ProPred, an epitope prediction algorithm (https://webs.iiitd.edu.in/raghava/propred/index.html). The predicted threshold was set to the default value of 3%. The prediction score was calculated using Propred and TEPITOPE (http://datamining-iip.fudan.edu.cn/service/TEPITOPEpan/index.html). Each peptide was synthesized to contain nine core amino acids, which were selected according to the AFP amino acid sequence. Peptide synthesis was performed using Mimotope (Melbourne, Australia) and purity ≥ 80% was confirmed using high-performance liquid chromatography.

### Preparation of peripheral blood mononuclear cells (PBMCs)

PBMCs were prepared using a method described previously^12^. Briefly, whole blood samples were collected from patients and healthy individuals, and PBMCs were isolated by centrifugation using Lymphoprep (AXIS-SHIELD PoC AS, Oslo, Norway). The isolated PBMCs were resuspended in RPMI 1640 (Gibco, Grand Island, NY) containing 10% heat-inactivated fetal bovine serum (FBS), 100 U/mL penicillin, and 100 μg/mL streptomycin. Fresh PBMCs were used for proliferation assays and unused PBMCs were stored frozen at −80 °C until use.

### Enzyme-linked immunospot (ELISPOT) analysis

For ELISPOT analysis, 96-well plates (MultiScreen; Merck Millipore, Cork, Ireland) were coated with the anti-human IFN-γ antibody (Mabtech, Nacka, Sweden). The antibody was fixed by overnight incubation at 4 °C. The following day, each well was washed four times with phosphate-buffered saline (PBS) and blocked with RPMI 1640 containing 5% FBS for 2 h at 25 °C. Using two wells for each peptide, 10 μg/mL of the peptide and 3 × 10^5^ PBMCs were added to each well and the mixture was cultured in RPMI 1640 containing 5% FBS. After a 24 h incubation period for the PBMCs with peptides, the plates were washed eight times and 100 μL of the biotin-labeled anti-human IFN-γ antibody was added and the plates were further incubated overnight at 4 °C. After washing the plates four times, streptavidin AP (Mabtech) was added to each well and the plates were incubated for 2 h. After washing the plates four times with PBS, freshly prepared NBT/BCIP solution (Biorad, Hercules, CA) was added. The plates were washed with distilled water to stop the reaction and dried at room temperature. The number of specific spots was calculated by subtracting the number of spots in a well without peptides from the number of spots in a well with peptides. The reaction was considered positive when >10 specific spots were calculated and there were more than twice the number of spots in the wells with peptides than in those without peptides. The negative control wells contained only PBMCs in the medium, whereas the positive control wells contained 10 ng/mL phorbol 12-myristate 13-acetate (Sigma, St. Louis, MO, USA).

The reactivity of the PBMCs against the peptides after the depletion of CD8^+^ and CD4^+^ cells was evaluated by ELISPOT, as described above. The determination of the depletion of the CD8^+^ and CD4^+^ cells was performed by labeling the PBMCs with MicroBeads (Miltenyi Biotec, Auburn, CA) followed by the use of a magnetic separator.

### Proliferation assays

For proliferation assays, 10 μg/mL of each peptide and 2 × 10^5^ freshly prepared PBMCs were added to each well of a 96-well plate and cultured in RPMI 1640 containing 10% FBS. After a 5 day culture period, 1 μCi/mL ^3^H-thymidine was added to each well and the cells were cultured for a further 24 h. The contents of each well were then transferred onto a 96-well filter plate using a cell harvester, and the radioactive uptake by the cells in each well was measured using a liquid scintillation counter. With regard to the stimulation index, the results were considered positive when the mean value of a well containing a target peptide was more than twice the mean value of a negative control well. The negative control wells contained only PBMCs and medium, while the positive control wells contained 10 ng/mL phytohemagglutinin (PHA).

### Peptide-binding assays

An immortalized EBV-infected human B-lymphocyte cell line (kindly provided by RIKEN BRC through the National Bio-Resource Project of MEXT, Japan), expressing HLA class II molecules, was used for the peptide-binding assays as HLA class II-expressing cells. The immortalized human cells were cultured in RPMI 1640 containing 20% FBS. The immortalized human cells and fluorescein isothiocyanate (FITC)-labeled peptides (1 mg/mL) were inoculated in each well of a 96-well plate and incubated for 2 h. Each well was washed twice with PBS, and the cells were analyzed using flow cytometry. The wells containing FITC-labeled preS1 (23–33)^20^ (a peptide derived from the hepatitis B virus, which is an HLA-DRB1*0405-restricted epitope) were used as control wells, while those containing the immortalized human cells without the addition of a peptide were used as the negative control. In addition, the leukemia cell line K562 was used as a negative control.

### Blocking assays

Blocking assays using the HLA antibodies were evaluated by ELISPOT. In brief, 10 μg/mL of the HLA-ABC (W6/32; BioLegend, Dan Diego, CA), HLA-DP (BRAFB6: Santa Cruz Biotechnology, Dallas, TX), HLA-DQ (SPV-L3: AbD Serotec, Oxford, UK), and HLA-DR (L243: BioLegend, SanDiego, CA) antibodies was added to the wells containing PBMCs and peptides for co-culture. The wells containing PBMCs alone or PBMCs with a peptide were used as controls.

### Statistical analysis

Univariate analyses comparing the background factors of the T cell response positive and negative groups against the AFP-derived epitopes were performed using the Mann–Whitney *U*-test and chi-square test. Cause-specific survival (CSS) estimates were calculated using the Kaplan–Meier method. *P*-values <0.05 were considered statistically significant.

## Results

### Patient profiles

Clinical data from the HCC patients who participated in this study are summarized in Table 1. During blood collection, 36 patients had HCC and 11 patients had a history of HCC treatment, with median serum AFP levels of 27 and 11 ng/mL, respectively. Of the 36 HCC patients, 11 had a primary tumor >2 cm in diameter, while the remaining patients had a primary tumor of ≤2 cm in diameter. In addition, 24 patients had multiple tumors with ≥ 2 nodes, while the remaining patients had a solitary tumor. Based on the Union for International Cancer Control TNM classification, 8, 16, 5, and 7 patients had stage I, II, III (A/B), and IV (A/B) tumors, respectively.

### Selection of potential MHC class II-restricted peptides derived from AFP

ProPred was used to predict the amino acid sequences. The amino acid sequence for the MHC class II allele was predicted from the entire set of HLA-DRB1 epitopes selected by the online service. In total, there were 14 types of peptides, of which 3 were for pre-existing peptides. Therefore, the remaining 11 types were selected from the peptides predicted to bind to multiple DRB1 alleles within the amino acid sequence, followed by the selection of additional peptides for HLA-DRB1*0405 (13.4% of the Japanese population^21^), *1502 (10.6% of the Japanese population), *1501 (7.7% of the Japanese population), *1302 (5.9% of the Japanese population), and *0101 (5.8% of the Japanese population), which occur at high frequencies in the Japanese population (Table 2). The prediction score of each peptide was calculated using ProPred and TEPITOPE.

**Table 2:** Peptides.

Peptide	Source	Start position	Amino acid sequence	Prediction score (ProPred)			Prediction score (TEPITOPE)		
				DRB1 *0405	DRB1 *0901	DRB1 *1502	DRB1 *0405	DRB1 *0901	DRB1 *1502
AFP_1_	AFP	1	MKWVESIFLIFLLNFTESRT	4.4500		5.6000	2.4264	2.814	5.5083
AFP_22_	AFP	22	HRNEYGIASILDSYQCTAEI	2.9000		3.6500	3.0698	2.0349	3.6029
AFP _62_	AFP	62	EVSKMVKDALTAIEKPT	2.0000		1.8000	0.3903	−0.3918	1.7524
AFP _134_	AFP	134	ASIPLFQVPEPVTSC	1.0000		1.2000	0.9899	0.943	1.2312
AFP _155_	AFP	155	RETFMNKFIYEIARRHPFLY	1.2000		2.8000	1.3064	2.6014	2.7413
AFP _169_	AFP	169	RHPFLYAPTILLWAARYDKII	3.7800		3.7600	3.2598	2.7795	3.6407
AFP _210_	AFP	210	TKELRESSLLNQHACAVMKN	0.1000		0.9800	0.1937	0.3807	0.998
AFP _224_	AFP	224	CAVMKNFGTRTFQAITVTKLSQ	3.6000		2.2000	2.7082	2.0703	2.145
AFP _246_	AFP	246	KFTKVNFTEIQKLVLD	ND		1.5000	−0.4778	0.82	1.3595
AFP _346_	AFP	346	EKNIFLASFVHEYSRRHPQL	2.6000		0.7000	2.6849	1.3533	0.7363
AFP _361_	AFP	361	RHPQLAVSVILRVAKG	ND		1.4500	−0.909	−1.1422	1.3422
AFP _416_	AFP	416	CGLFQKLGEYYLQNAFLVAYT	1.4500		2.8000	1.2723	3.2562	2.7063
AFP _428_	AFP	428	QNAFLVAYTKKAPQLTSSELM	2.1000		1.9000	2.4694	1.5843	1.8786
AFP _549_	AFP	549	KQEFLINLVKQKPQITEEQ	0.6000		ND	1.7001	1.0736	0.0559
preS1	HBV preS	23	GFFPDHQLDPA	ND		0.7000	−1.3916	−1.9603	0.6149

AFP134, AFP246 and AFP361 contain the previously published epitopes. “preS1” is the previously published epitope.

ND, no data.

### PBMC response to AFP-derived epitopes

**Figure 1:**
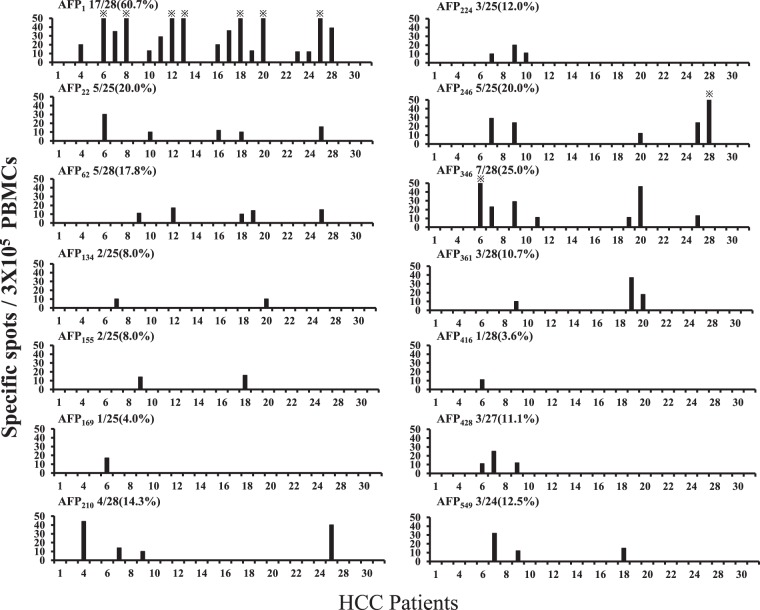
Analysis of peripheral blood T cell responses to AFP-derived epitopes. The number of spots on IFN-γ ELISPOT is shown. The response was determined to be positive when >10 specific spots were calculated and there were more than twice the number of spots in wells with peptides than in those without peptides. The amino acid sequence of each peptide is shown in Table 2. *indicates >50 spots.

An IFN-γ ELISPOT assay was performed to evaluate whether PBMCs from the 28 HCC patients reacted to the synthesized peptides. The peptides that showed positive reactivity in >20% of the patients were AFP_1_, AFP_22_, AFP_246_, and AFP_346_, with reaction rates of 60.7%, 20.0%, 20.0%, and 25.0%, respectively (Fig. 1). In contrast, the peptides that showed positive reactivity in <20% of the patients were AFP_62_, AFP_134_, AFP_155_, AFP_169_, AFP_210_, AFP_224_, AFP_361_, AFP_416_, AFP_428_, and AFP_549_. Among all the peptides, AFP_1_ showed the highest positive reaction rate for PBMCs and had the highest number of wells with >50 spots.

Similarly, the reactivity of PBMCs collected from the 14 healthy individuals to the synthesized peptides was evaluated using IFN-γ ELISPOT. The proportion of PBMCs showing positive reactivity tended to be lower than that of the PBMCs collected from the HCC patients. The peptides AFP_1_, AFP_246_, AFP_346_, and AFP_361_ showed positive reactivity in >20% of the healthy individuals; however, none of the wells with these peptides had a spot count >50 (Supplementary Fig. 1). Representative images of Fig. 1 and Supplementary Fig. 1 are shown in Supplementary Fig. 2.

### IFN-γ ELISPOT for CD8^+^ and CD4^+^ cell-depleted PBMCs

**Figure 2:**
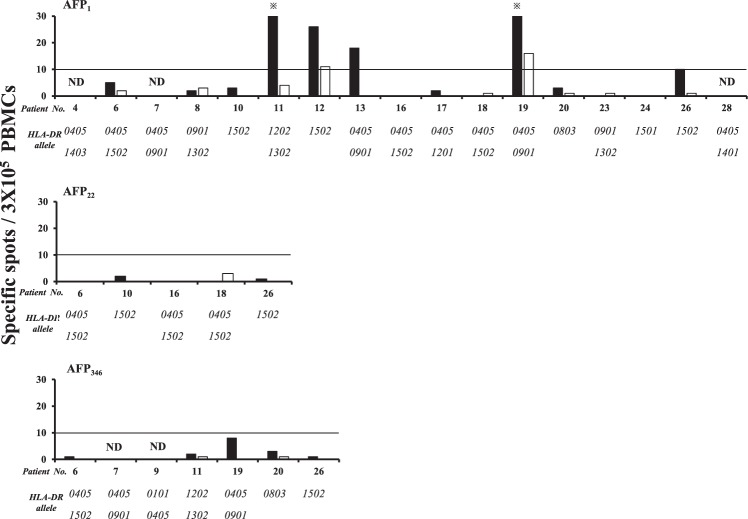
Responses of CD8^+^ and CD4^+^ cell-depleted PBMCs to AFP-derived epitopes. The numbers of spots on IFN-γ ELISPOT and the HLA-DR alleles of each patient are shown. Black and white bars denote the results for CD8^+^ and CD4^+^ cell-depleted PBMCs, respectively. The positive rate of CD8^+^ cell-depleted PBMCs against AFP_1_ was 35.7% (5/14), while that of CD4^+^ cell-depleted PBMCs was 14.3% (2/14). ND denotes “not determined”. *indicates >30 spots.

To confirm whether the lymphocytes that showed reactivity in the ELISPOT screening (Fig. 1) were CD4^+^ or CD8^+^ cells, MicroBeads were used to deplete CD8 and CD4 cells from the PBMCs, and IFN-γ ELISPOT was performed. The purity of the population was confirmed using flow cytometry (Supplementary Fig. 3). In all cases where CD8/4 was depleted, the fraction was validated by flow cytometry analysis. The peptides AFP_1_, AFP_22_, and AFP_346_, which showed a high rate of reactivity on ELISPOT, were used for this experiment (Fig. 2). The CD8^+^ cell-depleted PBMCs had a higher number of spots than the CD4^+^ cell-depleted PBMCs, indicating that the positive reactivity observed on ELISPOT (Fig. 1) was due to CD4^+^ cells. Representative images of Fig. 2 are shown in Supplementary Fig. 4.

In addition, analysis of the HLA-DR alleles of the patients whose PBMCs reacted positively after being depleted of CD8^+^ cells showed the presence of many HLA-DRB1*0405, *0901, and *1502 alleles, indicating that these HLA-DR alleles may have contributed to the observed reactions. The number of spots observed for AFP_1_, which showed the highest reactivity during the earlier screening by ELISPOT (Fig. 1), was also demonstrated to be high in the second ELISPOT (Fig. 2).

### Proliferation assays

**Figure 3:**
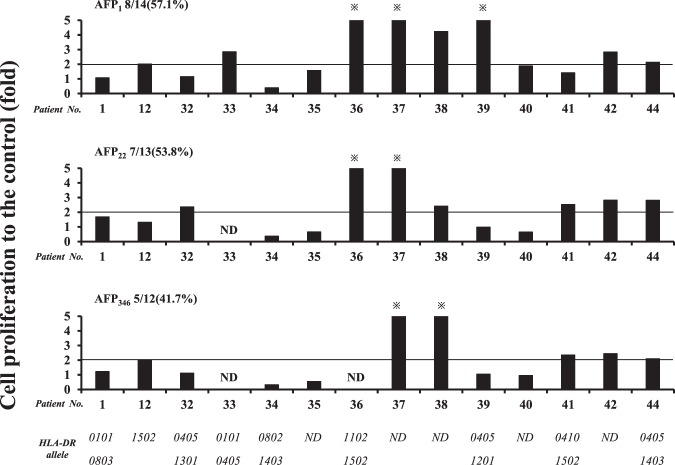
Analysis of the proliferative activity of PBMCs against AFP-derived epitopes. Multiple analyses comparing the control and the HLA-DR alleles of each patient are shown. Positivity was assigned when the stimulation index was more than twice that of the control. Positive frequencies for each peptide are shown. ND denotes “not determined”. *indicates >5.

Proliferation assays were performed to evaluate the reactivity of PBMCs to peptides according to the degree of cellular proliferation (Fig. 3). As shown in Fig. 2, the peptides used for these assays included AFP_1_, AFP_22_, and AFP_346_, which were the peptides that showed high reactivity in the screening by ELISPOT (Fig. 1). In addition to the PBMCs from patients used during the screening by ELISPOT, fresh PBMCs were collected from the HCC patients and those with a history of HCC treatment. All three peptides showed a positive reactivity of >40%, with AFP_1_ showing the highest positive rate. When the measured value was compared with the negative control, it was significantly higher for AFP_1_ and AFP_22_ (Supplementary Fig. 5). In several patients, the IFN-γ ELISPOT of PBMCs co-cultured with the peptide for 5 days was performed in the same manner as in the proliferation assays and the response was stronger than at the time of the screening ELISPOT (Supplementary Fig. 6). Therefore, we confirmed antigen-specific proliferation. Thus, the proliferation assays confirmed that the PBMCs reacted to the peptides. Furthermore, HLA-DRB1*0405 and *1502 were found to be present in the patients whose HLA-DR alleles could be analyzed.

### Binding assays

**Figure 4:**
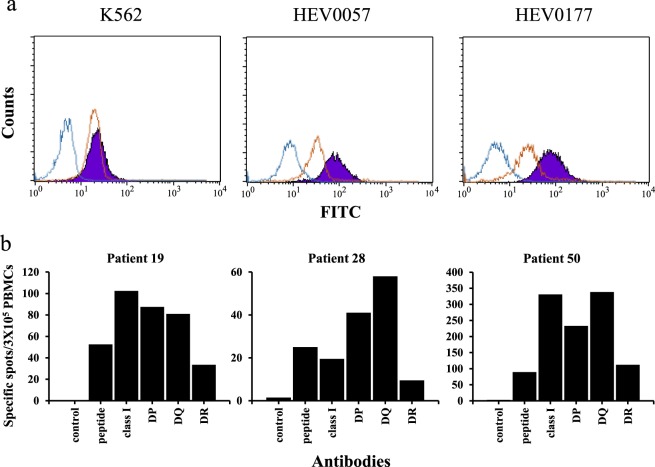
Analysis of the binding of labeled peptides to MHC molecules and the direct *ex vivo* analysis of PBMCs against AFP_1_ epitopes using an HLA antibody. (**a**) The affinity of each FITC-labeled AFP_1_ epitope and positive control peptides (preS1 23–32) to the human immortalized cells was measured using flow cytometry. HEV0057 and HEV0177 cells expressed homozygous HLA-DRB1*0901 and *0405, respectively. Control (without peptides), positive control peptide, and AFP_1_ are shown in blue, reddish brown, and purple, respectively. Each experiment was performed three times. (**b**) Direct *ex vivo* analyses (IFN-γ ELISPOT) of PBMCs against AFP_1_ were conducted using the HLA class I, HLA-DP, HLA-DQ, or HLA-DR antibodies. The numbers of spots are shown. Control well contains PBMCs only (without HLA antibody or peptides). Peptide wells contained peptide and PBMCs (without HLA antibody). Antibody wells contained PBMCs, peptides, and each antibody.

Binding assays were performed using AFP_1_, which was the peptide that showed the highest reactivity to PBMCs, in order to analyze its affinity towards HLA-DR molecules (Fig. 4a). Using FITC-labeled AFP_1_, the binding affinity of the peptide to cell lines expressing HLA-DR was analyzed by flow cytometry. Cell lines expressing homozygous HLA-DR molecules, including HEV0057 (HLA-DRB1*0901) (RRID:CVCL_T098) and HEV0177 (HLA-DRB1*0405) (RRID:CVCL_T166), were used. The K562 cell line, which does not express HLA molecules, was used as a negative control. FITC-labeled preS1 (23–33)^20^, an HLA-DRB1*0405-restricted epitope, was used as a control peptide. For the K562 cells, the fluorescence intensities of preS1 and AFP_1_ were at similar levels; however, for the HEV0577 and HEV0177 cells, the fluorescence intensity of AFP_1_ was stronger than that of preS1. These results confirmed the binding of AFP_1_ to the HLA molecules. Each experiment was performed three times on different days, with all three repetitions showing similar results. Representative data are shown.

### Blocking assays to confirm MHC restriction

To confirm the MHC restriction of AFP_1_, IFN-γ ELISPOT was conducted using MHC class I and class II antibodies (Fig. 4b). Using these antibodies, the binding of AFP_1_ to appropriate HLA molecules was shown to be inhibited and the type of HLA molecule contributing to the response was confirmed. The HLA-DR alleles of the HCC patients whose PBMCs were used were as follows: HLA-DRB1*0405/0901 for patient 19, HLA-DRB1*0405/1401 for patient 28, and HLA-DRB1*1501/1502 for patient 50. The production of IFN-γ was shown to be suppressed by the HLA-DR antibodies in patients 19 and 28. In patient 50, the production of IFN-γ was approximately the same with or without the antibodies. On the other hand, the class I antibodies HLA-DP and HLA-DQ did not suppress the production of IFN-γ in any patient. Thus, the binding of AFP_1_ to the HLA-DR molecules was confirmed.

### Clinical profiles of the patients who showed a positive or negative T cell response against AFP_1_

**Figure 5:**
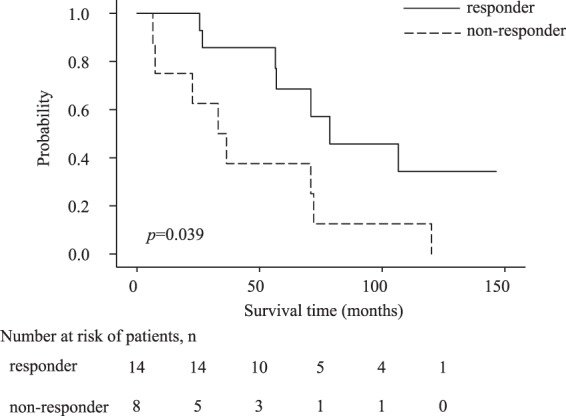
The Kaplan–Meier curve of cause-specific survival for death from liver cancer. Analysis of patients with stages II–IV HCC during blood collection. The relationship between duration (month) from the first day of HCC treatment and the cause-specific survival rate of death from liver cancer is shown using the Kaplan–Meier curve. A responder group that showed a T cell response against AFP_1_ and a non-responder group that did not show a response on ELISPOT using PBMCs or proliferation assays were compared.

To examine the characteristics of the HCC patients who showed an immune response against the AFP-derived MHC class II-restricted epitopes, the clinical profiles of these patients were analyzed. According to the IFN-γ ELISPOT (Fig. 1) and the proliferation assays (Fig. 3), the patients who showed a T cell response against the AFP_1_ peptide were classified into a positive group (n = 24), while those who did not show such a response were classified into a negative group (n = 16; Supplementary Table 1). Notably, the proportion of females tended to be significantly higher in the positive group than in the negative group (*p* = 0.07); however, no significant factors could be identified. In other words, the T cell response against AFP_1_ was induced regardless of tumor size, tumor multiplicity, the presence/absence of vascular invasion, or the etiology of liver disease. Next, the survival rates of the HCC patients between initial treatment and death due to HCC were analyzed to obtain cause-specific survival (CSS). When CSS was analyzed in the HCC patient group during blood collection, in which patients were determined to have clinical stage II, III, or IV HCC according to the TNM classification, the CSS of the positive group (n = 14) that showed a T cell response against the AFP_1_-derived epitope was significantly better than that of the negative group (n = 8), which did not show a response (*p* = 0.039; Fig. 5).

### T-cell response against AFP-derived epitopes before and after HCC treatment

**Figure 6:**
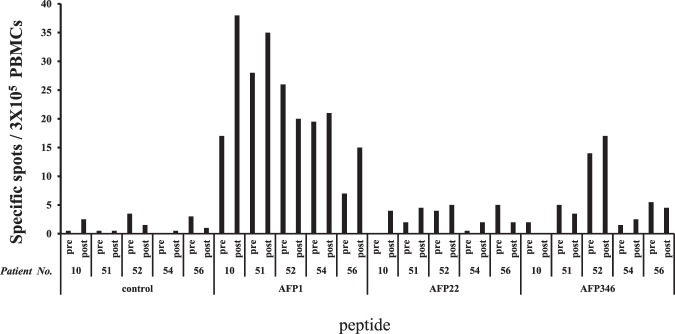
T cell response to MHC class II-restricted AFP-derived epitopes before and after HCC treatment. The numbers of spots obtained on IFN-γ ELISPOT, each peptide, and the patient number are shown. The control well contained only PBMCs (without peptides).

To examine changes in the T cell response against AFP-derived MHC class II-restricted epitopes by HCC treatment, peripheral blood samples were collected before and after HCC treatment from the patients who underwent radiofrequency ablation (RFA) (n = 4) and transcatheter arterial chemoembolization (TACE, which was a vascular embolization using anticancer drugs for HCC) (n = 1). PBMCs were collected within 3 to 42 days after treatment (Supplementary Table 2). Following the isolation of PBMCs, IFN-γ ELISPOT was performed on five patients whose peripheral blood was collected before and after the treatment, whereas the PBMCs used were prepared within the post-treatment 3 month period. The peptides used were AFP_1_, AFP_22_, and AFP_346_ (Fig. 6), which were the peptides that showed a high response rate from the screening by ELISPOT (Fig. 1). Representative images of Fig. 6 are shown in Supplementary Fig. 7. For AFP_1_, four of five patients showed an increased frequency of epitope-specific T cells after treatment. Further examination, according to the presence of specific HLA-DR alleles (Supplementary Table 2), revealed that all patients with the HLA-DRB1*1502 allele, the one patient with the HLA-DRB1*0405 allele, and one of the two patients with the HLA-DRB1*0901 allele had an increased frequency of epitope-specific T cells.

## Discussion

AFP is a cancer-related antigen unique to HCC and has been reported to be a possible therapeutic target for immunotherapy. While clinical trials of a cancer vaccine have been conducted using an AFP-derived class I-restricted epitope as the antigen, the clinical efficacy of AFP remains uncertain^10,11^. However, from these clinical trials, the activation of CD4^+^ T cells has been suggested to be required for sufficient levels of clinical efficacy.

We previously succeeded in identifying human telomerase reverse transcriptase-specific HTL epitopes using an epitope prediction algorithm^22^. Similarly, this website was used to predict AFP-derived MHC class II-restricted HTL epitopes in this study. Peptides were synthesized based on the amino acid sequences of the epitopes predicted to bind to various DRB1 alleles, and IFN-γ ELISPOT was conducted using PBMCs obtained from the HCC patients. The peptides AFP_1_, AFP_22_, AFP_246_, and AFP_346_ showed a response in >20% of the patients based on IFN-γ ELISPOT. Furthermore, IFN-γ ELISPOT using CD8^+^ cell-depleted PBMCs showed a high spot count; therefore, it was indicated that the response was due to CD4^+^ cells. Among the patients who showed a response on ELISPOT (Fig. 1), many did not continue to show a response on ELISPOT after the depletion (Fig. 2). This is likely due to differences in the timing of blood collection and the process used for depletion, which may have impacted the immune response. When the HLA-DR alleles of the patients who showed a positive response on IFN-γ ELISPOT were analyzed, the frequencies of the HLA-DRB1*0405, *0901, and *1502 alleles were high; therefore, it was indicated that these alleles contributed to the response. Among the synthesized peptides, AFP_1_ showed the highest response and a high number of spots on the IFN-γ ELISPOT performed after depletion of the CD8^+^ cells. According to previous reports on AFP-derived MHC class II-restricted HTL epitopes, the *ex vivo* detection of AFP-specific T cells in peripheral blood is difficult^23^. Thus, T cells induced by peptide stimulation are generally used for experiments. However, PBMCs were stimulated only overnight in this study, indicating that AFP_1_ is an epitope with the ability to respond directly *ex vivo*. These results indicate that the immunogenicity of the AFP_1_ epitope identified in this study was strong. These epitopes with high immunogenicity were able to be isolated in this study because the epitopes were preliminarily designed using a computer software program and the PBMCs were obtained from Japanese patients, in whom HLA is relatively homogeneous.

AFP_1_ contains several MHC class I-restricted subdominant epitopes, i.e., AFP_1_ contains subdominant restricted epitopes of HLA-A24^12^ and HLA-A2^24^. In fact, although low in frequency, 2 of 14 patients showed a positive response on IFN-γ ELISPOT using CD4^+^ cell-depleted PBMCs.

AFP-specific CD4^+^ cell reactions are observed at low frequency among healthy individuals^25^. However, AFP_1_ showed a positive response at a high frequency compared with other peptides (Supplementary Fig. 1). Therefore, AFP_1_ is an epitope that may have the potential to induce specific T cells among healthy individuals.

Furthermore, proliferation assays were performed to evaluate the response against peptides not only by IFN-γ production but also by the level of cellular proliferation. All three peptides analyzed showed a response rate >40%; thus, the T cell response against the peptides was also demonstrated by an increase in cellular proliferation. By examining the HLA-DR alleles of the HCC patients, we found that the patients possessed the HLA-DRB1*0405, *0901, or *1502 alleles. AFP_1_ showed a response at a high frequency even in proliferation assays. Therefore, we considered that AFP_1_ is an immunodominant epitope able to not only produce IFN-γ but also enhance cellular proliferation.

Next, the binding affinity of AFP_1_, which showed the highest response rate to T cells of the HCC patients, to HLA-DR molecules was examined using flow cytometry. As shown in Fig. 4a, AFP_1_ was confirmed to bind to human immortalized cells that were homozygous for the expression of HLA-DRB1*0405 or HLA-DRB1*0901. Furthermore, to examine MHC class II restriction, IFN-γ ELISPOT was performed in which the binding of AFP_1_ to each HLA molecule was inhibited by the respective HLA antibody. This effect of causing a decrease in IFN-γ production was higher with the addition of the HLA-DR antibody compared with that of other antibodies. These results indicated that AFP_1_ was able to bind to the HLA-DR molecules. The HLA-DR alleles of the patients used were *0405, *0901, and *1502 and it was indicated that AFP_1_ is a restricted epitope of these multiple alleles. Therefore, we considered that the strong immunogenicity of AFP_1_ was due to its ability to bind to multiple HLA-DR molecules.

The relationship between the clinical data of the HCC patients and the T cell response against the AFP-derived epitopes was analyzed. However, no significant differences in terms of serum AFP levels between the group that responded to the epitopes and the group that did not were observed. To initiate an immune response against cancer, antigen-presenting cells (APCs) must take up antigens from the tumors and an immune response must be triggered by the activation of T cells in response to the APCs^26^. In this study, AFP was the tumor antigen and was required for AFP-derived epitope-specific T cell activation. However, it has been reported that AFP reduces the ability of dendritic cells to stimulate T cell proliferation and inhibits the proliferation of CD8^+^ and CD4^+^ cells^27^. Thus, it is considered that T cell proliferation is suppressed when the serum AFP level is high. According to previous reports, the CD4^+^ T cell response against AFP is significantly high among HCC patients with low serum AFP levels and patients with early-stage HCC^17^. However, the CD4^+^ T cell response against AFP_1_ did not show a correlation with serum AFP levels or the clinical stage of HCC in this study. On the other hand, in patients with stage II-IV HCC, the CSS of the patient group that showed an immune response against AFP_1_ was better than that of the group that did not show such a response. These findings suggest that patients who exhibit a T cell response against AFP_1_ have a better prognosis even at an advanced clinical stage. In this context, our results indicated that AFP_1_ is an immunodominant epitope that may elicit an immune response in patients with high serum AFP levels or advanced HCC. Furthermore, having an immune response against AFP_1_, even at an advanced clinical stage, appeared to be beneficial for survival.

Next, changes in the immune response of patients before and after HCC treatment were examined by IFN-γ ELISPOT using the PBMCs obtained from the patients. Indeed, CD4 responses are known to increase after HCC treatment^18,22^. Our results showed that the number of responses against the AFP-derived epitopes increased in several patients, as reported previously. Therefore, an increase in the T cell response against AFP_1_ was indicated after HCC treatment. In contrast, there were patients in whom the T cell response decreased. As previously reported, immune responses are known to reach a peak 2–4 weeks after RFA treatment^28^. The sample from patient 52 was collected within 1 week after RFA treatment; therefore, the timing of sample collection may have influenced our results. Moreover, the involvement of dendritic cell activation associated with tumor cell death and heat shock proteins released after treatment is indicative of changes in the frequency of T cells specific to antigen epitopes after HCC treatment^29^. With regard to the decreased immune response after HCC treatment, a detailed mechanism has not been elucidated, thus further research on the underlying mechanism is warranted.

To the best of our knowledge, no reports of immunodominant HTL epitopes in AFP_1_ amino acid sequences have been published. In this study, AFP_1_ was indicated to be an immunodominant epitope capable of binding to multiple HLA-DR molecules. Therefore, immunotherapy using this epitope may be beneficial to HCC patients as part of a combination therapy with other immune therapies such as ICIs.

## Supplementary information

Supplementary information.

Supplementary information 2.
