# Supplementary material for: A novel α-fetoprotein-derived helper T-lymphocyte epitope with strong immunogenicity in patients with hepatocellular carcinoma
Source: Sci Rep. 2020 Mar 4;10:4021. doi: 10.1038/s41598-020-60843-4 (PMC7055302; doi:10.1038/s41598-020-60843-4)

Supplementary Figure 1

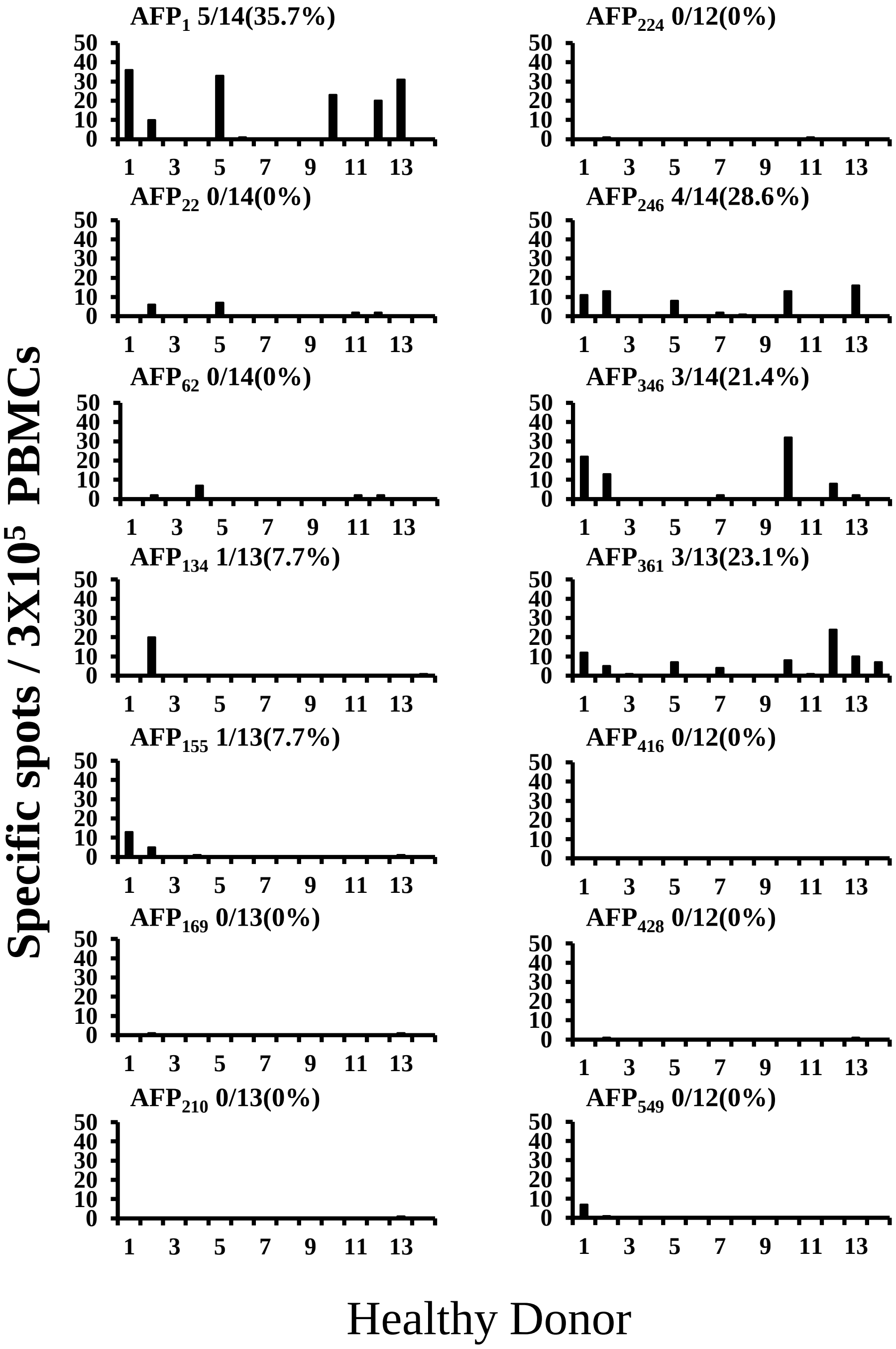

Supplementary Figure 2

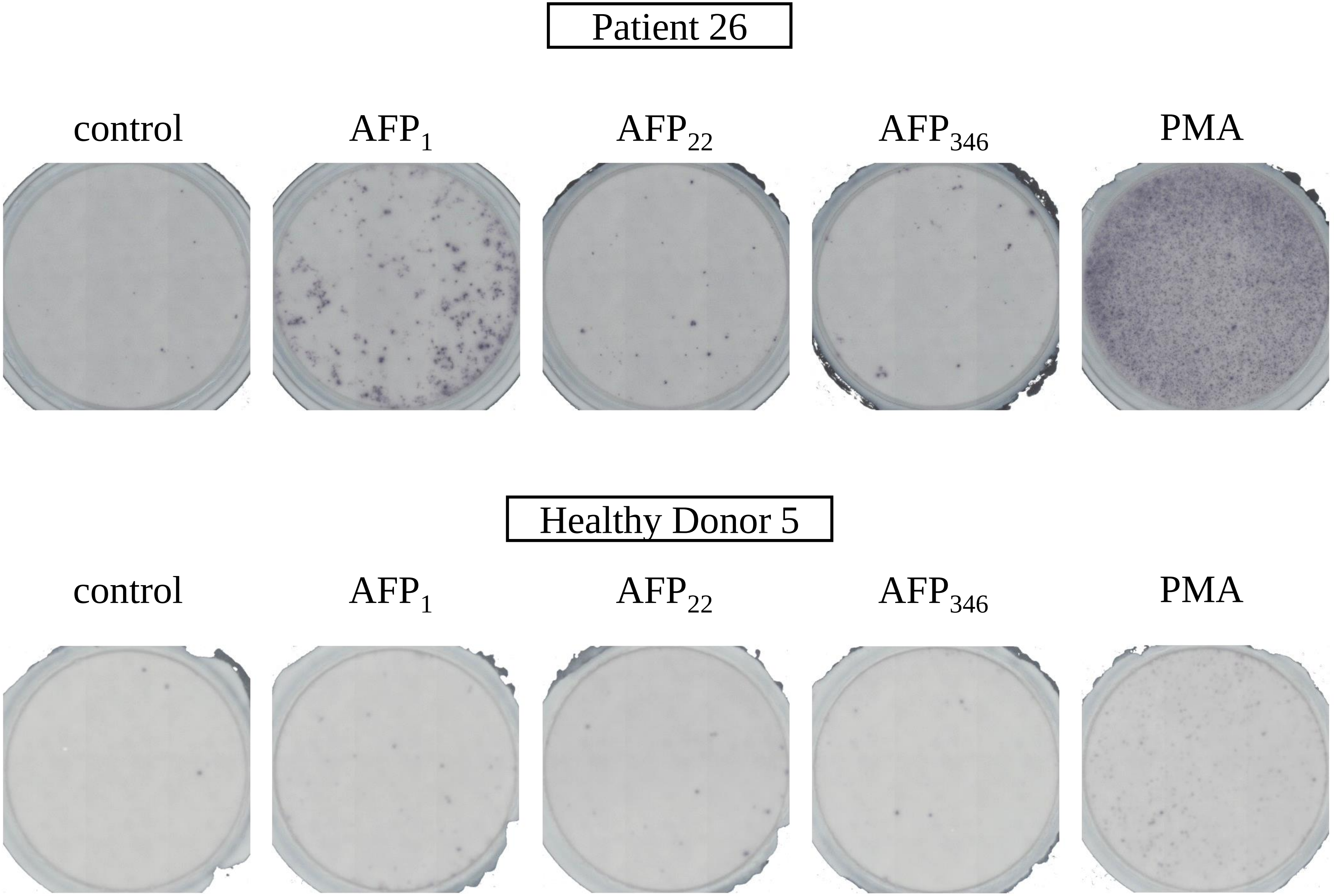

Supplementary Figure 3

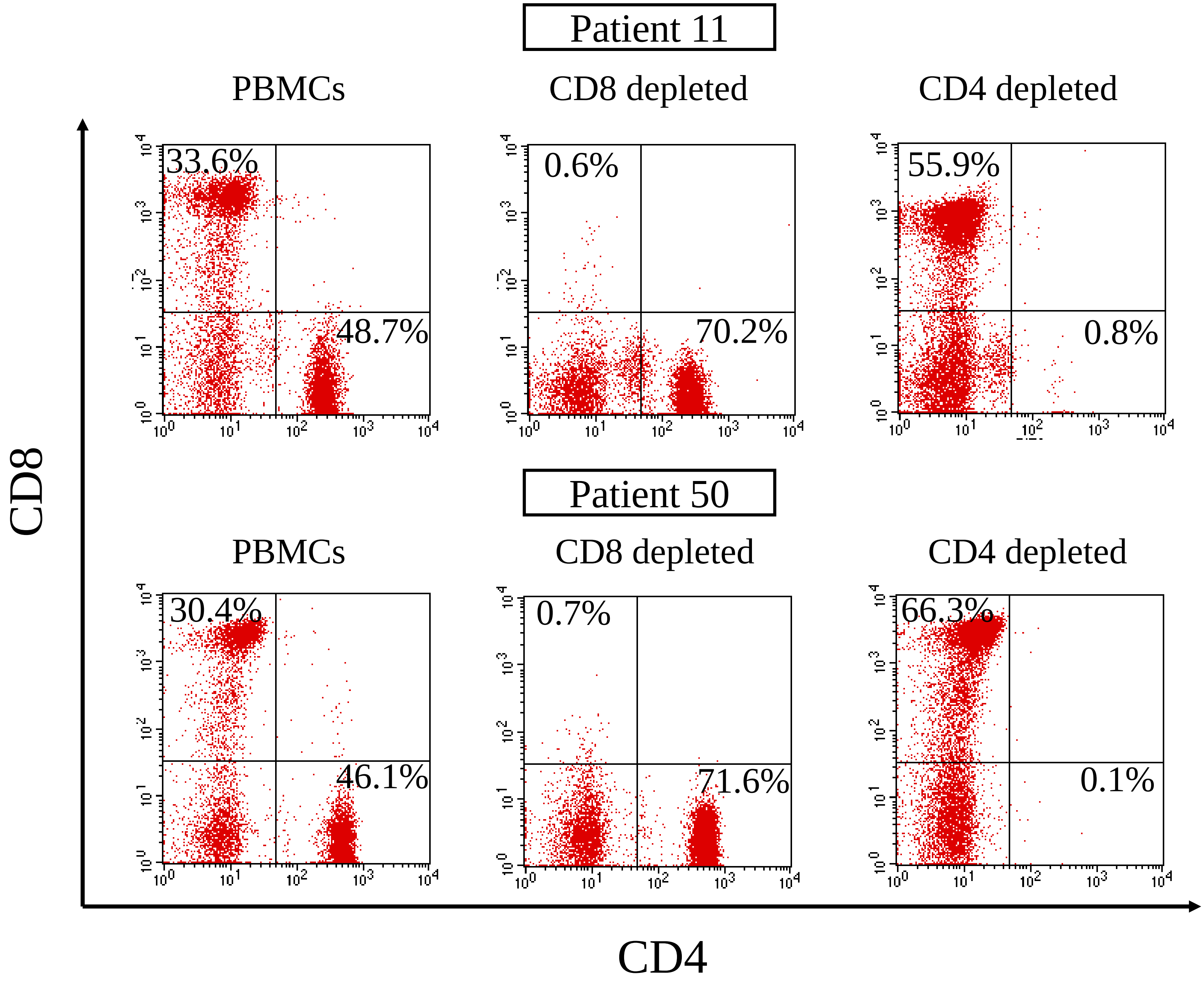

Supplementary Figure 4

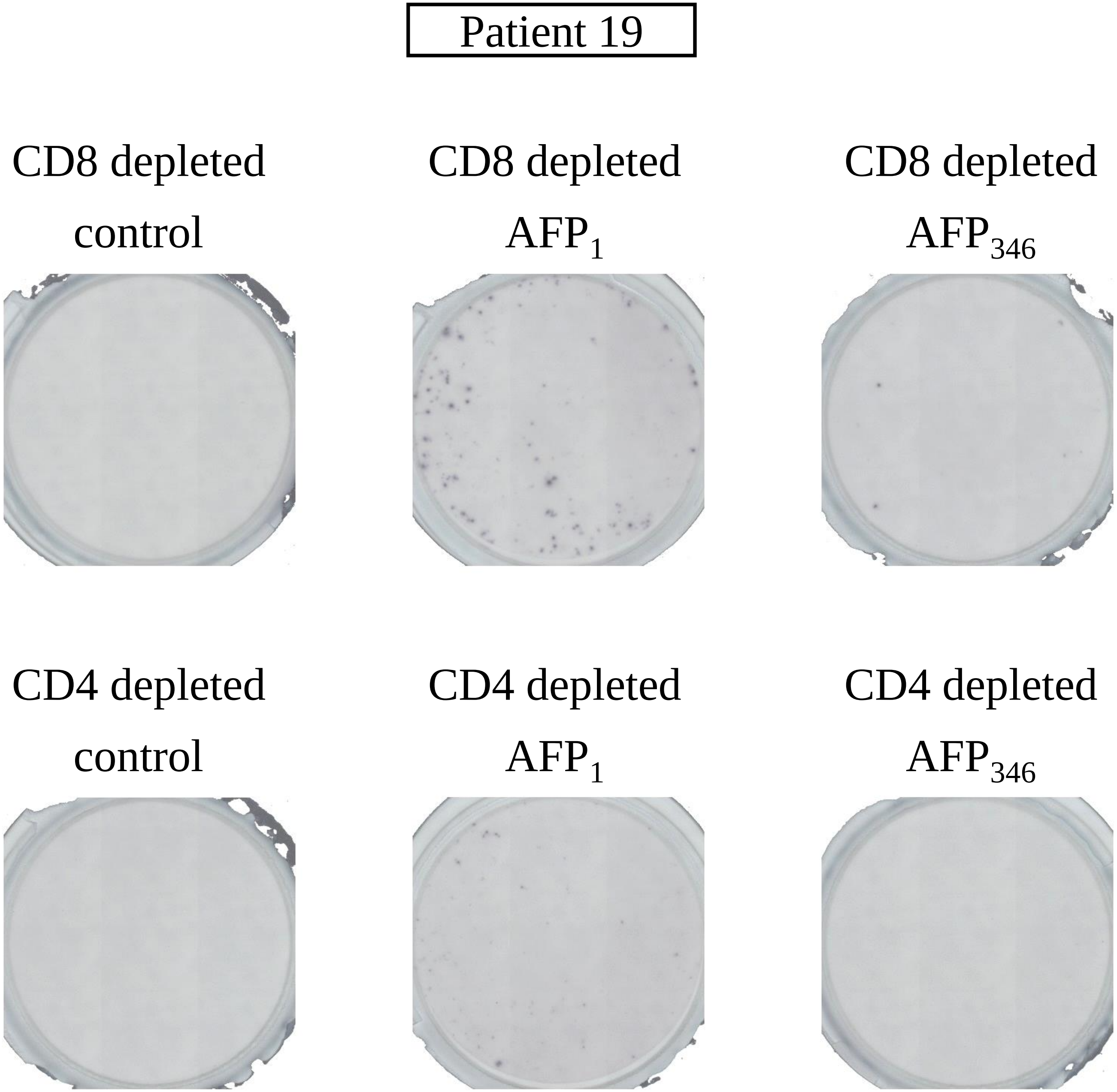

Supplementary Figure 5

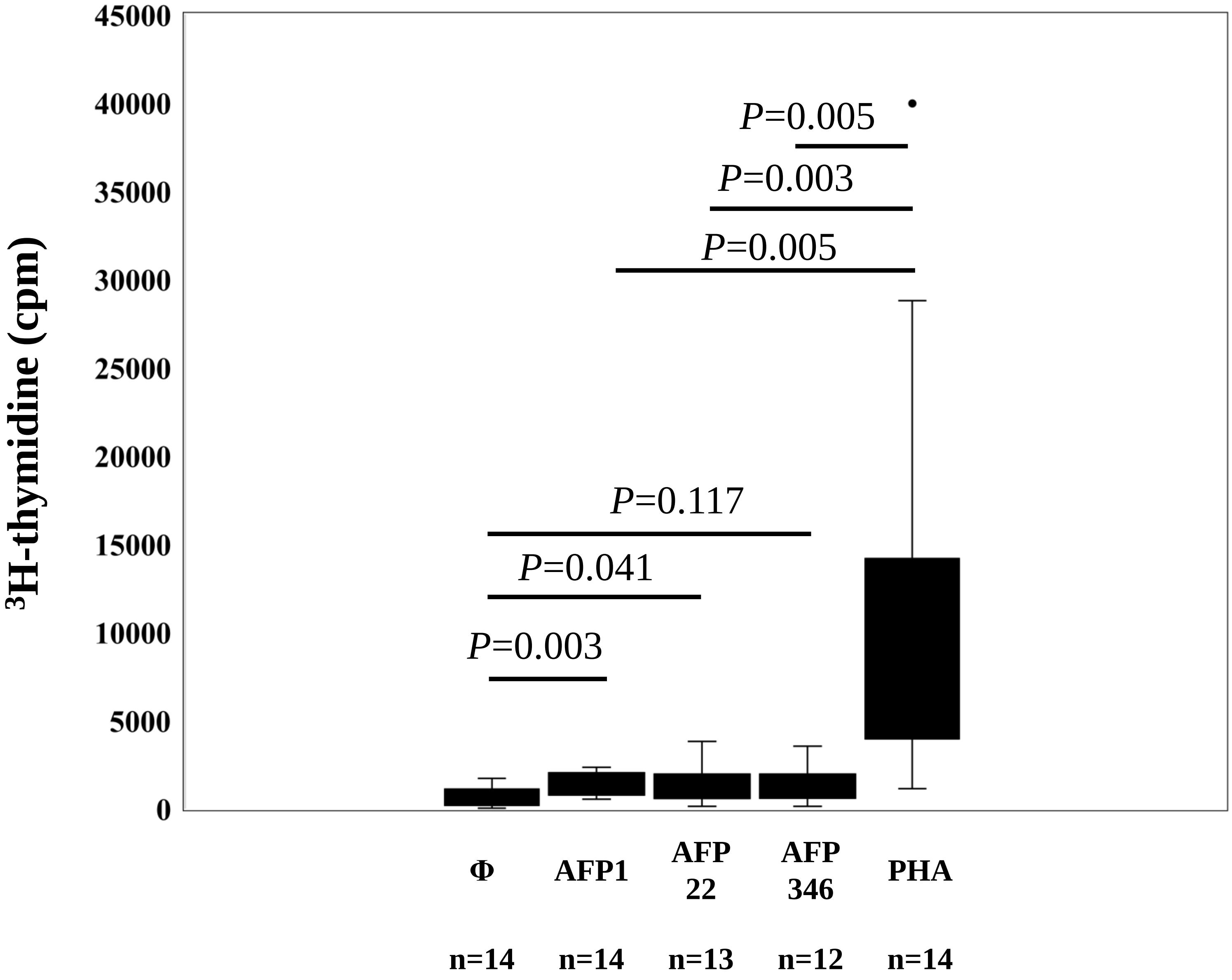

Supplementary Figure 6

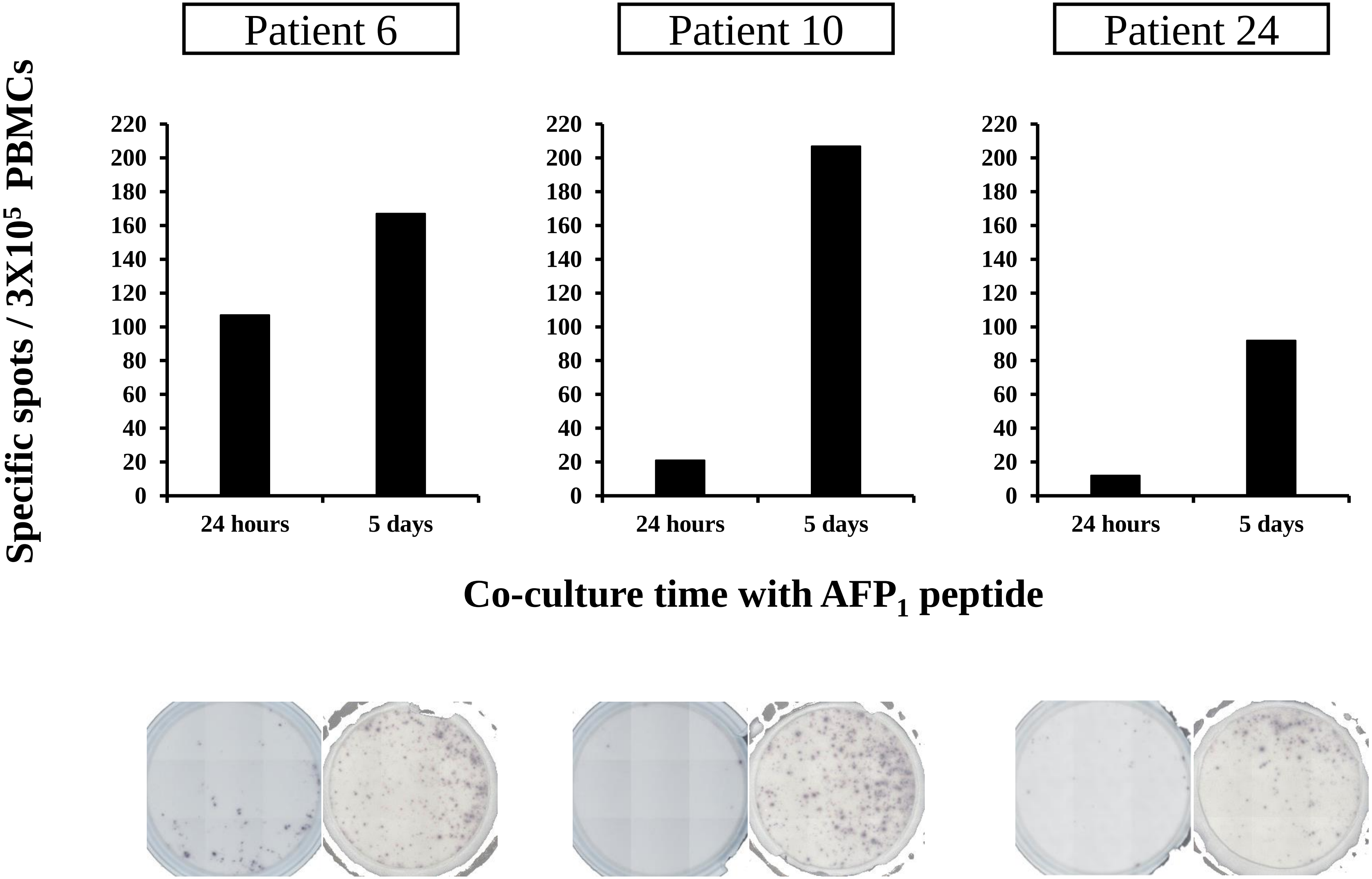

Supplementary Figure 7

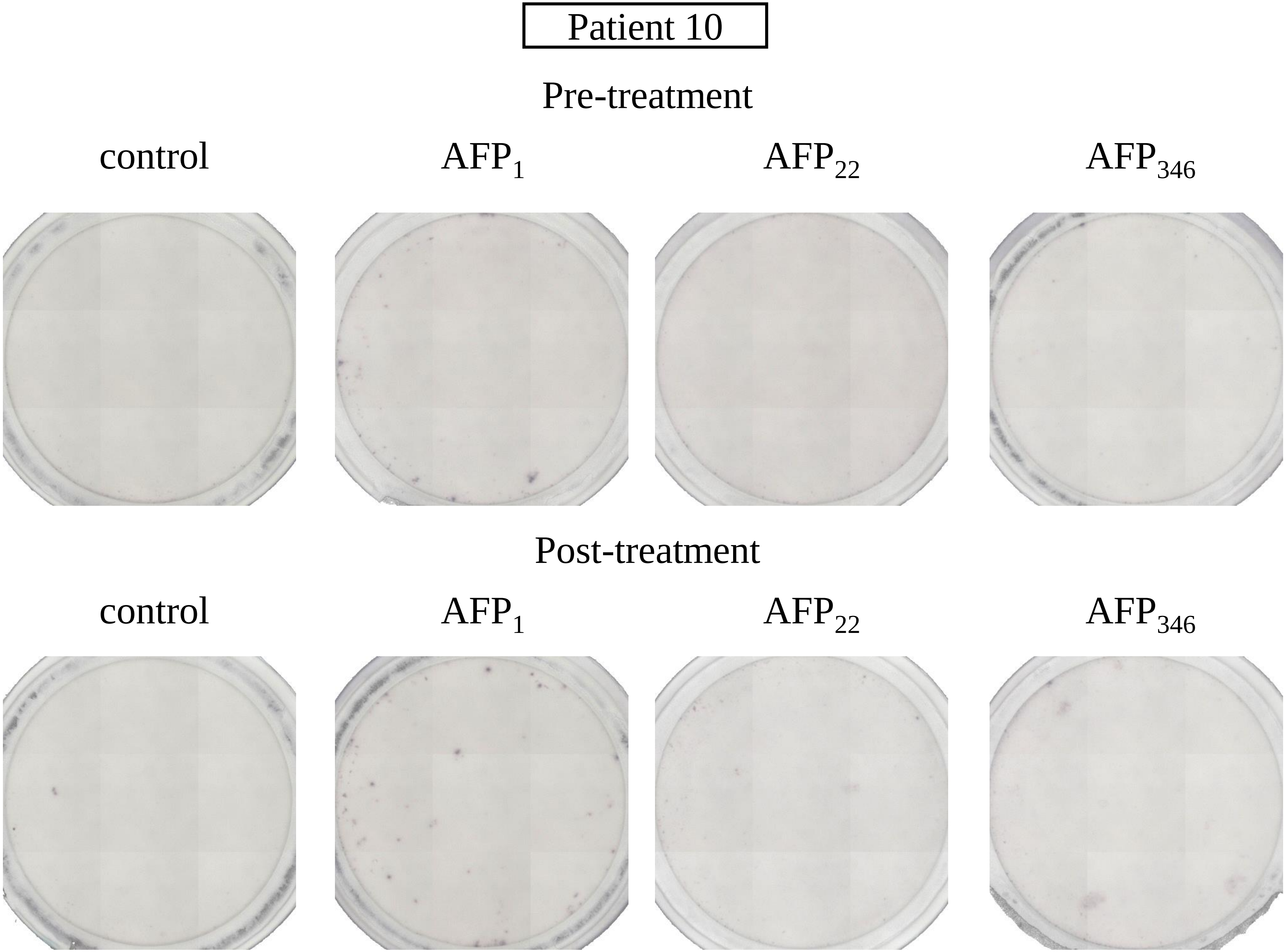

Supplement: Supplementary file 1 — Supplementary information. [file 41598_2020_60843_MOESM1_ESM.pdf]
