# Supplementary material for: A novel α-fetoprotein-derived helper T-lymphocyte epitope with strong immunogenicity in patients with hepatocellular carcinoma
Source: Sci Rep. 2020 Mar 4;10:4021. doi: 10.1038/s41598-020-60843-4 (PMC7055302; doi:10.1038/s41598-020-60843-4)
Supplement: Supplementary file 2 — Supplementary information 2. [file 41598_2020_60843_MOESM2_ESM.doc]

**A novel α-fetoprotein-derived helper T-lymphocyte epitope with strong immunogenicity in patients with hepatocellular carcinoma**

Toshikatsu Tamai, Eishiro Mizukoshi*, Masashi Kumagai, Takeshi Terashima, Noriho Iida, Masaaki Kitahara, Tetsuro Shimakami, Kazuya Kitamura, Kuniaki Arai, Taro Yamashita, Yoshio Sakai, Tatsuya Yamashita, Masao Honda, Kazumi Fushimi, and Shuichi Kaneko

Supplementary Table 1

Supplementary Table 2

Legend of Supplementary Figure 1

Legend of Supplementary Figure 2

Legend of Supplementary Figure 3

Legend of Supplementary Figure 4

Legend of Supplementary Figure 5

Legend of Supplementary Figure 6

Legend of Supplementary Figure 7

**Supplementary Table 1.** Univariate analysis of the effect of variables on the T cell response against AFP-derived peptides.

|  | T cell response positive  (n = 24) | T cell response negative  (n = 16) | *P* |
| --- | --- | --- | --- |
| Tumor bearing/previous treatment | 17/7 | 12/4 | 0.77 |
| Age, years (range) | 68.5 (54-81) | 67 (52-75) | 0.22 |
| Sex, male/female | 17/7 | 15/1 | 0.07 |
| Platelet count, ×10^4^/µL (range) | 9.2 (5.1-28.3) | 11.8 (7.1-22.6) | 0.21 |
| Active prothrombin, % (range) | 81 (56-97) | 83 (67-104) | 0.20 |
| ALT, IU/L (range) | 37 (10-98) | 37 (16-159) | 0.40 |
| Albumin, g/dL (range) | 3.8 (2.4-4.7) | 3.7 (3.3-4.6) | 0.75 |
| Total bilirubin, mg/dL (range) | 0.8 (0.4-1.8) | 1.0 (0.4-1.8) | 0.11 |
| AFP, ng/mL (range) | 20 (5-40550) | 13.5 (4-2912) | 0.73 |
| Main tumor size, mm (range) | 13 (4-83) | 19 (6-150) | 0.23 |
| Tumor　multiplicity  (multiple/solitary) | 12/5 | 6/6 | 0.26 |
| Vascular invasion, +/- | 3/14 | 2/10 | 0.95 |
| Clinical stage  (TNM I/II/III/IV) | 3/10/1/3 | 4/2/3/3 | 0.12 |
| Etiology  (HBV/HCV/HBV+HCV/NBNC) | 3/14/1/6 | 4/6/0/6 | 0.41 |

Values represent the median.

**Supplementary Table 2.** Supportive information about the HLA-DRB1 allele and treatments.

| Patient No. | HLA-DRB1 | Treatment | Days from treatment |
| --- | --- | --- | --- |
| 10 | 1502 | RFA | 19 |
| 51 | 0901,1502 | RFA | 42 |
| 52 | 0901,1301 | RFA | 3 |
| 54 | 0405,1502 | RFA | 18 |
| 56 | 0802,1502 | TACE | 25 |

TACE, transcatheter arterial chemoembolization; RFA, radiofrequency ablation

**SUPPLEMENTARY FIGURE LEGENDS**

**Supplementary Figure 1. Analysis of peripheral blood T-cell response to AFP-derived epitopes in healthy donors.** The numbers of spots obtained on IFN-γ ELISPOT are shown. Cases in which the number of spots was > 10 and the number of spots in wells with peptides was more than twice that in wells without peptides were considered positive. The amino acid sequence of each peptide is shown in Table 2.

**Supplementary Figure 2. Representative ELISPOT image of “Figure 1” and “Supplementary Figure 1”.** The ELISPOT figure of patient 26 and healthy donor 5 are shown.

**Supplementary Figure 3. Analysis of the fraction of CD8^+^ and CD4^+^ cells using flow cytometry.** CD8^+^ and CD4^+^ cell-depleted PBMCs were labeled with a fluorescent antibody and analyzed. The separation of CD8^+^ and CD4^+^ cells was confirmed.

**Supplementary Figure 4. Representative ELISPOT image of “Figure 2”.** The ELISPOT figure of patient 19 is shown.

**Supplementary Figure 5.** **The proliferation of T cells upon peptide stimulation.** Values from the proliferation assay using ^3^H-thymidine were summarized for each peptide. We performed the Student’s *t*-test to determine if there were statistically significant differences among the negative control peptides, PHA (positive control), and each treatment peptide (AFP1, AFP22, AFP346).

**Supplementary Figure 6. Enhanced T cell response after 5 days culture with peptide.** The numbers of spots obtained on IFN-γ ELISPOT are shown. PBMCs from several patients were incubated with the peptide for 5 days in the same manner as the proliferation assays. The T cell responses were then compared to those cultured for 24 hours as shown in Figure 1. The representative ELISPOT images of each are shown below the graph.

**Supplementary Figure 7. Representative ELISPOT image of “Figure 6”.** The ELISPOT figure of patient 10 is shown.
